# Gold nanocrystals with DNA-directed morphologies

**DOI:** 10.1038/ncomms12873

**Published:** 2016-09-16

**Authors:** Xingyi Ma, June Huh, Wounjhang Park, Luke P. Lee, Young Jik Kwon, Sang Jun Sim

**Affiliations:** 1 Department of Chemical & Biological Engineering, Korea University, Seoul 136713, Republic of Korea; 2 Department of Electrical, Computer & Energy Engineering, Materials Science & Engineering Program, University of Colorado, Boulder, Colorado 80309, USA; 3 Institute of Quantitative Biosciences & Biophysics, Departments of Bioengineering, Electrical Engineering & Computer Science, University of California Berkeley, Berkeley, California 94720, USA; 4 Departments of Pharmaceutical Sciences, and Chemical Engineering & Materials Science, University of California Irvine, Irvine, California 92697, USA; 5 Green School, Korea University, Seoul 136713, Republic of Korea

## Abstract

Precise control over the structure of metal nanomaterials is important for developing advanced nanobiotechnology. Assembly methods of nanoparticles into structured blocks have been widely demonstrated recently. However, synthesis of nanocrystals with controlled, three-dimensional structures remains challenging. Here we show a directed crystallization of gold by a single DNA molecular regulator in a sequence-independent manner and its applications in three-dimensional topological controls of crystalline nanostructures. We anchor DNA onto gold nanoseed with various alignments to form gold nanocrystals with defined topologies. Some topologies are asymmetric including pushpin-, star- and biconcave disk-like structures, as well as more complex jellyfish- and flower-like structures. The approach of employing DNA enables the solution-based synthesis of nanocrystals with controlled, three-dimensional structures in a desired direction, and expands the current tools available for designing and synthesizing feature-rich nanomaterials for future translational biotechnology.

## Abstract (web summary)

Bottom-up synthesis of colloidal metallic nanomaterials with a designable structure is challenging. Here, the authors report the directed crystallisation of gold by a single DNA molecular regulator, using it to synthesise gold nanocrystals with defined complex morphologies.

## Graphical abstract

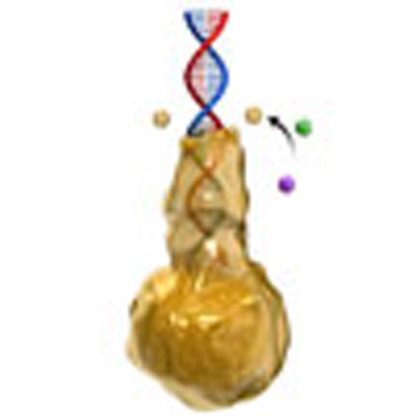

Preparation of metallic nanomaterials with a desirable shape is of great interest and remains a critical technological gap^1^,^2^,^3^,^4^. In contrast to the molecular assembly of discrete objects using organic templates (for example, DNA origami)^5^,^6^,^7^,^8^,^9^, the bottom-up synthesis of metallic nanomaterials with a designable structure at high resolution is challenged by difficulty in manipulating atoms that are transient in nature^10^,^11^. Seed-coating agents such as polymers, surfactants and synergetic ions are frequently employed to vary the growth of seed facets with different surface energy levels^4^,^12^; however, the generating nanostructures are limited to be highly symmetric with identical surface facets^2^,^13^,^14^,^15^. A metallization strategy of using a pre-designed template, such as DNA and DNA origami, in overcoming the limitations has been investigated^16^,^17^,^18^,^19^,^20^. Recently, single-stranded DNA (ssDNA) with varying sequences has also been used to synthesise nanoparticles with different morphologies^21^,^22^,^23^,^24^,^25^,^26^. All attempts interceding in the morphological evolution of nanomaterials demonstrated limited success to control the crystallization of nanomaterials in desired structures. The control of crystallization in a desired direction, which allows the synthesis of metallic nanomaterials with a higher control resolution but has not been fully demonstrated, can be achieved *via* directed, controlled crystallization along molecular regulators. DNA is known for encoding molecular information, guiding the directional synthesis of RNA and its complementary pairing between strands, and also enabling highly specific multi-dimensional assemblies. In this study, we design double-stranded DNA molecules (dsDNA) anchored onto gold nanoseed (AuNS) to regulate the crystallization of Au atoms from the AuNS surface to the DNA terminus, mainly driven by the distribution gradient of gold precursors (HAuCl_4_). This strategy promises a highly facile preparation of nanometals with DNA contour-following shape- and size-controlled architectures in an aqueous environment.

## Results

### Evidence of DNA-directed crystallization

**Figure 1:**
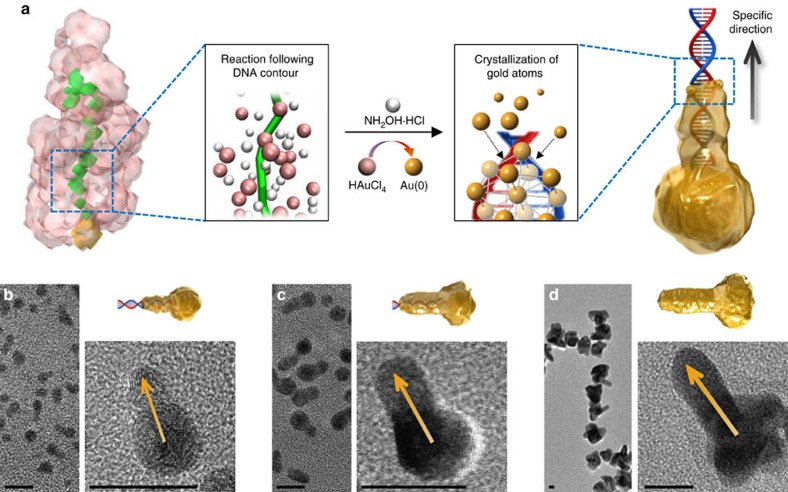
Directed crystallization of gold by dsDNA. (**a**) Scheme showing DNA-directed crystallization of a AuNC. (**b**–**d**) TEM and HR-TEM images of the crystalline nanostructures. Scale bars, 10 nm.

We hypothesized that the redox reaction between HAuCl_4_ and a reducing agent (NH_2_OH·HCl) in aqueous solution, that is, the gold crystallization, could be directed by DNA contour resulting from the dynamics of water at the DNA interface^27^,^28^ (Fig. 1a). One linear dsDNA (L1; 30 base pairs) conjugated on the AuNS, AuNS-L1, resulted in one-directional growth of gold nanocrystals (AuNCs), as shown in the transmission electron microscopy (TEM) and high-resolution TEM (HR-TEM) images (Fig. 1b–d; control experiments shown in Supplementary Fig. 1). The gold crystallization along the DNA (∼10 nm in length) appeared faster than that on the nanoseed surface (∼5 nm in diameter; Fig. 1b). Once the branch was developed, the steady crystallization on both the branch and the nanoseed formed the branched AuNC shape^29^ (Fig. 1c,d). The dimensions of the branch in the AuNC directed by the dsDNA (Fig. 1b–d) were tunable in ranges of 1.3±0.7–25.1±3.0 nm in length and 2.4±0.3–10.9±1.1 nm in cross-sectional diameter, simply by adjusting HAuCl_4_ concentrations (Methods section and Supplementary Fig. 2). The control level achieved in this study is comparable to the latest advance in bottom-up fabrication of colloidal nanostructures^13^,^30^.

### Mechanism of DNA-directed crystallization

**Figure 2:**
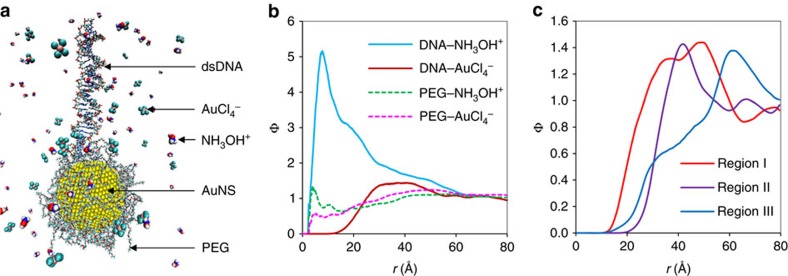
All-atom molecular dynamics simulation. (**a**) Snapshot image of a simulated AuNS-L1 system. (**b**) Radial distributions of ion species (NH_3_OH^+^, AuCl_4_^−^) around ligand species (DNA, PEG). (**c**) Radial distributions of AuCl_4_^−^ around three subgroups of the single dsDNA: Region I (bp index 1–10), Region II (bp index 11–20) and Region III (bp index 21–30), respectively, where bp index of the full dsDNA strand runs from 1 (the first bp having terminus anchored to AuNS) to 30 (the last bp containing a free terminus).

The process of the directional crystallization makes this approach fundamentally distinct from the conventional approaches in creating DNA-templated metallic nanostructures, where negatively charged DNA or DNA origami is patterned on a solid substrate, followed by isotropically interacting with positively charged metal precursors^20^ (Supplementary Note 1). In this approach, the growth of AuNCs was directed by dsDNA anchored onto AuNS (stabilized with polyethylene glycol (PEG) on the surface^31^) *via* reaction between two oppositely charged species of reaction sources (AuCl_4_^−^, NH_3_OH^+^) in the presence of a mildly acidic electrolyte at pH 5. An atomistic molecular-level insight on the spatial distributions of AuCl_4_^−^ and NH_3_OH^+^ ions around AuNS-L1 in aqueous media was provided by an all-atom molecular dynamics (MD) simulation (Methods section), as shown in a representative snapshot image (Fig. 2a). The simulation accounted for bonded and non-bonded interactions between ionic and molecular species but did not take into account chemical reactions between them. A computed radial distribution function (Φ) of the ion species (NH_3_OH^+^, AuCl_4_^−^) at a distance *r* from ligand species (DNA, PEG; Fig. 2b) revealed the dsDNA was surrounded by an NH_3_OH^+^ ion-concentrated inner shell (*r*=∼8 Å) and a rather distant outer AuCl_4_^−^-rich space (*r*=25–50 Å). In comparison, the distributions of both AuCl_4_^−^ and NH_3_OH^+^ at the vicinity of PEG-stabilized AuNS were relatively plateaued regardless of distance, except small spiked peaks near the surface. The simulation suggests that most redox reactions happened at the dsDNA/AuNS conjugation region where AuCl_4_^−^ ions around AuNS tend to interact with NH_3_OH^+^ ions, which are highly concentrated around dsDNA. In addition, AuNSs lower the activation energy required for AuCl_4_^−^ to reduce to Au atoms^32^. Herein, AuNS has dual functions of enriching the distribution of AuCl_4_^−^ and catalysing the redox reactions (Supplementary Fig. 3). Radial distribution functions of AuCl_4_^−^ ions around three sub-regions of the dsDNA, I (1–10 bps), II (11–20 bps) and III (21–30 bps) from the conjugation point to the terminus show that the concentration of AuCl_4_^−^ ions around DNA becomes higher when closer to the AuNS (Fig. 2c). The gradient of AuCl_4_^−^ availability in the direction along the dsDNA reflects the change in electrostatic environment by which AuCl_4_^−^ ions experience less electrostatic repulsion from negatively charged DNA backbone when they move toward PEGylated AuNS. The reactant pairs, AuCl_4_^−^ and NH_3_OH^+^ ions, are likely to encounter each other in the dsDNA/Au boundary which moves along the DNA contour as the gold crystallization proceeds, explaining the directional specificity. The dsDNA's contour renders a direction-specific guide in the synthesis of colloidal nanomaterials, while the conventional seed-coating agents are empirically selected, rather than logically designed, to produce symmetric nanostructures by tuning the energy difference of selected crystallographic facets of nanoseeds^4^,^12^.

**Figure 3:**
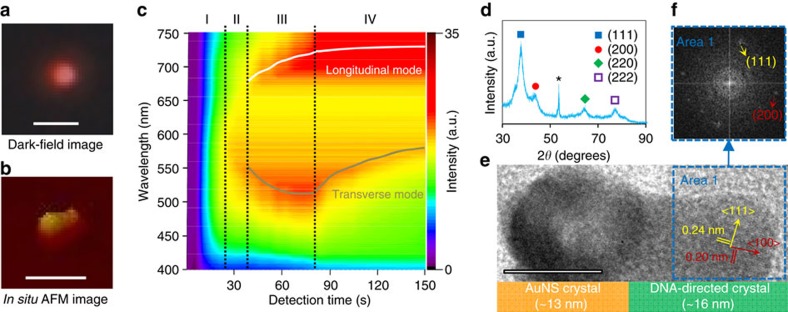
Investigations of the directed crystallization. Experimental verifications of AuNS-L1 directed AuNC by (**a**) dark-field microscopy and (**b**) AFM. Scale bars, 1 μm for dark-field image and 100 nm for AFM image. (**c**) Representative real-time Rayleigh scattering profile of single dsDNA-directed crystallization of AuNC. (**d**) X-ray diffraction spectrum of single dsDNA-directed AuNCs (*peak from silicon substrate). (**e**) HR-TEM analysis of crystallization along DNA. Scale bar, 10 nm. (**f**) FFT image of the selected ‘Area 1' in the HR-TEM image shown in **e**.

We further manifested the dsDNA-directed nanocrystal growth in single-nanoparticle resolution using a real-time, *in situ* nanoplasmonic sensing system (Fig. 3a–c and Supplementary Fig. 4). The AuNS with one dsDNA-directed nanocrystal growth changed colours from green to red in the dark-field images, while bare AuNC (that is, without dsDNA-directed nanocrystal) showed green colour throughout the entire imaging process (Supplementary Fig. 4b). The dynamic Rayleigh light-scattering spectra obtained by the system quantitatively revealed the dsDNA-directed evolutionary phases of nanocrystal growth (Fig. 3c). They include reduction phase I (no peak), recrystallization phase II (one peak), emerging phase III (two peaks) and ripening phase IV (two peaks). At Phase I, AuNS was too small to generate significant localized surface plasmon resonance signals during gold ions reduction to atoms. Recrystallization of gold atoms at Phase II increased the resonant Rayleigh scattering intensity at single *λ*_max_∼570 nm. As the two orientations of the nanostructure emerged (one in a perpendicular direction (transverse mode, *λ*_max_480–600 nm) and the other one in a parallel direction (longitudinal mode, *λ*_max_>670 nm) with respect to the electromagnetic field of incident light), the localized surface plasmon resonance bands split into two modes at Phase III. Noticeably, the longitudinal plasmon band red-shifted with the directional nanocrystal growth from the AuNS to the DNA terminus, while the transverse resonance blue-shifted in agreement with Gans' theory^3^. The growth was terminated in Phase IV with red-shifted transverse peaks attributed to an increase in particle diameter. Crystallographic investigation of the AuNCs by X-ray diffraction showed that the structure is predominantly bounded by {111} facets with the lowest surface energy (γ_111_<γ_100_<γ_110_; Fig. 3d)^4^,^15^. The analysis of the DNA-directed part of the AuNC confirmed crystal planes with spacing distances of around 0.20 and 0.24 nm (Fig. 3e,f), corresponding to <100> and <111> growth directions for an fcc structure of gold, along and across to the axial direction of DNA, respectively.

### Application of DNA-directed crystallization

**Figure 4:**
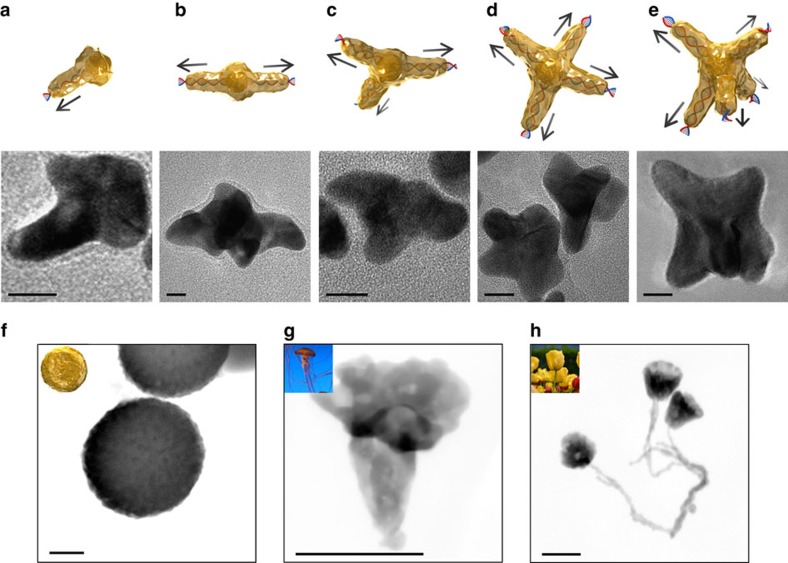
DNA-directed crystallization of AuNCs. (**a**–**e**) Illustrations and TEM images of star-shaped AuNCs with controlled branches from 1 to 5, respectively. Scale bars, 10 nm. (**f**) Disk-like, (**g**) Jellyfish-like and (**h**) Flower-like AuNCs taken by high-angle annular dark-field scanning TEM (HAADF-STEM). Scale bars, 100 nm.

We explored the feasibility of directional specific synthesis of AuNCs with various morphological features that are specifically programmable with the number and the topologies of DNA molecules, to meet the current demands for novel metallic nanomaterials in nanophotonics and nanosensors. First, star-shaped AuNCs were synthesized as they exhibit unusual nanoplasmonic properties, depending on detailed geometric parameters (for example, number of arms). The number of nanocrystal arms was molecularly dictated by the number of linear dsDNA (30 bps) grafted onto 5 nm-diameter AuNS (AuNS-L*n*, where *n*=1 through 5) using the ‘one-after-another' conjugation method; that is, DNA sequentially binds onto AuNS with each round of conjugation, and each anchor-position of DNA is self-regulated by electrostatic repulsions and steric hindrances^33^ (Supplementary Fig. 5). AuNCs in asymmetric pushpin-, water caltrop- and ‘T'-like shapes were obtained after DNA-directed nanocrystal growth from AuNS-L1, AuNS-L2 and AuNS-L3, respectively. AuNCs in two distinct shapes were synthesized using AuNS-L4: four-armed starfish- and tetrapod-like AuNCs. Similarly, five-armed AuNCs were formed with AuNS-5DNA (Fig. 4a–e and Supplementary Fig. 6). The DNA-directed AuNCs in various morphologies were statistically analysed for their yields in desired morphology, core diameter, branch length and branch diameter (Supplementary Table 1). We further conducted optical simulations using the commercial software COMSOL (ref. 34) to understand the plasmon behaviour of the AuNCs (Supplementary Note 2). The tunable plasmon hybridizations between the core and the branches suggest the high potential of harnessing the AuNCs in a defined shape as ‘optical barcodes' for a variety of applications such as label-free biodiagnosis^35^. The same synthetic approach using ssDNA did not yield AuNCs with multiple arms (Supplementary Fig. 7), indicating ssDNA failed to regulate the directional nanocrystal growth. We attribute this to the flexible nature of ssDNA (persistence length ∼2 nm)^36^ and the chemisorption of the exposed bases onto the AuNS surface^37^.

We also expressed the structural information of non-linear dsDNA into nonconventional AuNCs. The AuNSs (∼2.5 nm) first modified to bear amino groups were conjugated with 1-pyrenebutanoic acid succinimidyl ester (intercalator molecule). The planar aromatic pyrene portion of the intercalator then intercalated with the paired bases of dsDNA *via* the collective interactions of π–π stacking, van der Waals force and hydrophobic interactions^38^,^39^, causing the AuNSs to anchor in an average distance equivalent to 15 bps along the non-linear DNA as analysed by ImageJ (Supplementary Fig. 8). An open-circular DNA (846 bps) resulted in AuNCs with a disk-like, biconcave shape with tiny tips around the edges (termed D-AuNCs, Fig. 4f and Supplementary Fig. 9). Disk-shaped nanostructures manifested efficient *in vivo* transport^40^; remarkably, the D-AuNCs can generate much higher scattering intensities than lithographic nanodisks^41^ or wet-etching nanoplates^42^ in the near-infrared window (Supplementary Fig. 9h,i), implying promise as a superior optical contrast agent in biological tissues. Furthermore, we used plasmids with more complicated DNA topology in the directional crystallization strategy. The supercoiled plasmid pUC18 (2,686 bps) took on a structural transformation (Supplementary Fig. 10), and thereby regulated the growth of AuNC in jellyfish-like shape (Fig. 4g and Supplementary Fig. 11). Using a larger plasmid pBR322 (4,361 bps), interestingly, resulted in a flower pattern emerging (Fig. 4h and Supplementary Fig. 12). Noticeably, the long curving and adjacent DNA strands direct the formation of ‘stalks' of the nanoflower by the DNA-directed crystallization strategy, while DNA strands near each other undergo inter-strand interference and merge into a whole bulge structure in conventional DNA-templated approaches such as DNA mineralisation^20^. The regulation of the metallic crystallization process in desired directions with single DNA molecules is a key to unlocking designable features of bottom-up synthesis of colloidal nanometals.

## Discussion

Rapid progress in structural DNA nanotechnology^43^ will help to further expand the current method of designing and synthesizing feature-rich nanomaterials for many user-specified purposes. DNA-templated metallization using cationic metal ions produces either sequential necklaces or aggregative granular objects with a poorly controlled structural precision. In bottom-up synthesis, manipulating crystallization of atoms toward a guided direction is more facile in structurally controlling the morphology of nanomaterials than metallizing molecular templates where the enlargement of metal clusters simply magnifies the initial shape of templates. The control of nanomaterial crystallization *via* seeded growth with coating agents could be improved when organic molecules specifically coating targeted crystal facets of nanoseeds are employed in producing nanomaterials with desired nanostructures^4^,^12^. However, lacking general strategies for identifying organic molecules that are capable of coating a targeted crystal facet has been a key challenge^2^. dsDNA with a diameter of 2–3 nm, controllable length of 3.4–3.6 nm per turn, high structural rigidity (persistence length ∼50 nm) and well established modifications to anchor it onto a nanoseed, is an ideal molecular guide in directed crystallization of nanocrystals.

As demonstrated in the all-atom MD simulations, Au crystallization is initiated by NH_3_OH^+^ attracted to negatively charged dsDNA, seemingly non-specific to DNA sequence. Therefore, anionic polymers conjugated on the AuNS surface could theoretically direct the crystallization like DNA. Furthermore, according to the MD simulation, the anionic metal precursor on average tends to be away relatively far from DNA due to electrostatic repulsion; however, it should be noted that there is a gradient of the precursor's availability in the direction along DNA, from the AuNS conjugation-point to the terminus of DNA. This gradient is essential for the directional specificity of Au crystallization and therefore we speculate that the material diversity can be expanded if other metal precursors and reductants have a similar level of intricacy, precision and superior functions as the AuCl_4_^−^–DNA–NH_3_OH^+^ system. In the same synthetic system, programmable DNA nanostructures such as DNA nanotubes^44^,^45^, DNA origami^5^,^6^,^7^,^8^, DNA jigsaw^46^ and DNA brick^47^,^48^ can be utilized for directed crystallization of metallic nanomaterials. In contrast, the growth of silver using Ag^+^ did not follow the direction of DNA but resulted in rough-surfaced spherical nanocrystals due to its electrostatic property difference from AuCl_4_^−^ (Supplementary Fig. 13).

The morphological yield of the DNA-directed asymmetric nanocrystals is superior to the strategy used in a recent study to cast asymmetric shapes using pre-defined DNA molds^13^. The somewhat limited control over the AuNC's morphology was attributed to the competition of crystallization occurring along DNA (kinetically controlled regime)^49^,^50^ versus on the nanoseed (thermodynamically controlled regime)^12^,^15^. While the growth of a DNA-directed branch can be manipulated, the crystallization on the nanoseed is driven by the thermodynamic tendency of lowering surface energy, which is beyond exogenous control. Therefore, relatively limited control over the growth of the nanoseeds did not allow for 100% control over the morphology of the resultant structure of AuNCs. To maximize the exposed portion of dsDNA from the nanoseed surface, while allowing minimally required absorption of charged molecules^51^, low molecular weight PEG was used to form a monolayer on the seed, promoting the dsDNA to impart directionality during Au crystallization.

DNA's molecularly tunable structure and self-assembly makes it arguably the most widely used biomacromolecule in designing novel materials^43^. This study reports a utility of DNA as a molecular regulator of nanocrystal growth in a sequence-independent manner, enabling synthesis of metallic nanocrystals of desired shapes (synthesis-with-design) for a number of applications in biomedical imaging, optical antennas and biomolecular sensors. Directed by linear dsDNA, star-shaped AuNCs (nanostars) can be synthesized with single or multiple branches. Previous work on nanostar synthesis by using surfactants has shown that nanostars offer more optical hot spots than polyhedral nanocrystals because the nano branches provide natural focusing of electromagnetic field, and lead to higher sensitivity for nanoplasmonic applications (for example, biosensing in diagnosis)^22^,^52^,^53^,^54^,^55^,^56^,^57^,^58^. However, the conventional nanostars have uncontrolled branched structures and usually generate one broad absorbance peak, although few nanostars in a size of ∼100 nm or larger showed two peaks. Such a large structural polydispersity prohibits nanoplasmonic applications that take full advantage of the opportunities that nanostar geometry provides. Distinctively, DNA-directed nanostars offer a significant advantage to facilely tune the local electromagnetic field with their controllable branches (Supplementary Figs 14–16 and Supplementary Note 2), generating distinct absorbance spectra with two or three peaks. With conjugation to various probes (for example, antibodies), these nanocrystals can be utilized for simultaneous capture and quantification of multiple targets (for example, antigens).

## Methods

### DNA-directed synthesis of nanocrystals

Gold precursor (HAuCl_4_, 0.03%) and reductant (NH_2_OH·HCl, 1 mM) were separately dissolved in water and the pH of each solution was adjusted to be 5 (±0.1) by gradually adding NaOH under nitrogen environment. After the HAuCl_4_ aqueous solution was stirred and stabilized for 7 days at 4 °C, 250 μl of 2 nM linear DNA-conjugated AuNS or 0.5 nM plasmid DNA-conjugated AuNS was mixed with 100 μl of NH_2_OH solution and 57 μl of HAuCl_4_ solution, and a colour change was observed within one minute. After 15 min, the synthesized particles were rinsed by repeated centrifugation and re-suspension in water. By varying the amounts of gold precursor (3.8 μl, 9.4 μl, 28.3 μl) and reductant solution (6.7 μl, 16.6 μl, 50.0 μl, respectively), the size of the nanocrystals was controlled during the course of DNA-directed nanocrystal synthesis.

### All-atom molecular dynamics simulation

All-atom MD simulations were performed for a model system of AuNS-L1 in an aqueous solution containing AuCl_4_^−^ and NH_3_OH^+^ ions. The AuNS (3,925 Au atoms, diameter ∼5 nm) conjugated with dsDNA (30 bps) and 56 PEG chains (each with 18 monomeric units) *via* –S(CH_2_)_3_-linker at the 3′-terminus of dsDNA and –S(CH_2_)-linker at the PEG terminus, respectively. The remaining termini of dsDNA and PEG were capped with hydroxyl and methyl groups, respectively. A simulation box of 170 Å × 170 Å × 200 Å, which contains 40 AuCl_4_^−^ and 80 NH_3_OH^+^ ions as well as 1 AuNS-L1 and 333,056 water molecules, was generated with a periodic boundary condition. The CHARMM force field was used for AuNS-L1 where the force field parameters for Au, dsDNA, PEG and alkyl thiol were taken from the reported works^59^,^60^,^61^,^62^, while the CHARMM-compatible force fields for ion source were generated using ForceField Toolkit^63^ module implemented in VMD (ref. 64). With this system constitution, a NPT-ensemble MD simulation was carried out using NAMD2 program^65^. The temperature and the pressure were maintained at 298 K and 1 bar using the Langevin piston Nose–Hoover method^66^. A full system periodic electrostatics was employed by the particle-mesh Ewald method with a 1 Å grid spacing^67^. The cutoff and switching distances for van der Waals force were set to be 12 and 10 Å, respectively. The bonds involving hydrogen were constrained to be rigid by using the SHAKE algorithm^68^. The MD system was equilibrated for 3 ns with a 2 fs time step and the structure analysis was done for simulating a further 5 ns run with recording every 2 ps.

### Conjugation of AuNSs with linear DNA

All glassware used in the experiments were cleaned in freshly prepared aqua regia solution and rinsed thoroughly with ultra-pure water (18.2 mΩ ·cm^−1^) before use. Five hundred microlitres of monoavidin-coated magnetic particles (MPs) were first rinsed and reactivated by following the manufacturer's protocols (Bioclone). The MPs were then suspended in the conjugation buffer (Ocean NanoTech) and mixed with 500 μl of 100 μM 30-bp linear dsDNA1 (all oligonucleotides used in this study were purchased from Integrated DNA Technologies; sequence information is provided in Supplementary Table 2). After incubation for 60 min at 37 °C with gentle shaking, the mixture was placed on the magnetic separator (Ocean NanoTech) and the supernatant was removed. The collected particles were rinsed twice with the washing buffer (Ocean NanoTech), re-suspended in 600 μl of the hybridization buffer (Sigma-Aldrich), and mixed with 550 μl of 100 μM 30-bp linear dsDNA2 for 30 min at 37 °C with gentle shaking. The thiol-modified dsDNA2 was pre-treated with immobilized TCEP disulfide reducing gel by following the manufacturer's instructions (Thermo Scientific). Five minutes after adding 50 μl of the quenching buffer (Ocean NanoTech), the particles were collected using the magnetic separator under nitrogen. The resulting particles were washed twice by a hybridization wash pack (Sigma-Aldrich) and mixed with AuNS (5 nm, British BioCell International) at the equivalent molar ratio to DNA. Before conjugation with DNA, the AuNS coated with bis(p-sulfonatophenyl)phenylphosphine dihydrate dipotassium (BSPP, 100 ml of AuNS solution mixed with 100 mg BSPP) was purified three times by Microsep centrifugal devices (Pall Life Sciences, MWCO 30 K, 3,000*g*) and re-suspended in 50 ml of 100 mM cetylpyridinium chloride solution. For AuNS to be conjugated with the thiol groups of DNA, the mixture of MP–DNA and AuNS was incubated for 60 min at room temperature. The resulting MP–DNA–AuNS conjugates were collected using the magnetic separator, while free AuNS was thoroughly rinsed off with PBS buffer for three times. The conjugates were finally re-suspended in 500 μl of the elution buffer (2 mM D-biotin, 2 mM SH-PEG, 20 mM PBS; pH 7.4) to free AuNS conjugated with DNA strands (AuNS–DNA). The AuNS–DNA solution was purified by centrifugal filtration. The fabrication process for the first round yielded AuNS bound to one DNA strand (AuNS–1DNA). A second round was performed with AuNS–1DNA to add another strand. The resultant AuNS with two strands (AuNS–2DNA) were used to initiate a third round to make an AuNS with three DNA strands (AuNS–3DNA) and so on. Each fabrication cycle adds one DNA molecule conjugated onto AuNS (Supplementary Fig. 5). The procedures in the first round were repeated in the second and later rounds with slight modification. Before the mixture of the AuNS–DNA solution with MP–DNA, 50 μl of 50 mM desthiobiotin (Life Technologies) was added to the MP–DNA solution to block free monoavidin on the MP surface. The AuNS–DNA was stored in TE buffer (10 mM Tris, 0.1 mM EDTA; pH 8.0) at 4 °C for long-term storage. AuNS conjugated with ssDNA (30-bp linear ssDNA) was prepared using a similar method to that of AuNS conjugated with dsDNA. Both 5′ and 3′ ends of ssDNA were modified to be conjugated with MPs and AuNS, respectively. Due to its relatively low stability, ssDNA-conjugated AuNS was used immediately after the fabrication process.

### Intercalation of AuNSs in plasmid DNA

Equivalent amounts (5 mmol) of 3-bromopropylamine hydrobromide and 1-methylimidazole were added to 12.5 ml of ethanol and mixed under nitrogen for 24 h, followed by recrystallization by ethyl acetate. The resulting white powder, dried overnight in a vacuum desiccator, was dissolved in water to yield 5 ml of 200 mM ionic-liquid, followed by addition of 1 ml of 25 mM aqueous HAuCl_4_ while stirring to yield 2.5 nm AuNS. After 12 h, the precipitate collected by centrifugation was repeatedly rinsed with water and dried overnight at 60 °C under vacuum. The AuNS was re-suspended in 9 ml of water and then the solution was mixed with 1 ml of the mixed solution of 5 mM 2-{2-[2-(2-{2-[2-(1-mercaptoundec-11-yloxy)-ethoxy]-ethoxy}-ethoxy)-ethoxy]-ethoxy}-ethylamine hydrochloride and 5 mM SH-PEG with a volume ratio of 1:6 in ethanol, to form PEG-terminated self-assembled monolayer on the AuNS surface. After washing by centrifugal filtration (MWCO 10 K, 3,000*g*) three times with water, 250 μl of the resulting AuNS solution at 1 μM was mixed with 2 μl of 100 μM 1-pyrenebutanoic acid succinimidyl ester (intercalator; Life Technologies) for 30 min to have the amino groups of the self-assembled monolayer react with the succinimidyl ester groups of the intercalator. Then the solution was centrifuged, the supernatant was decanted and the pellet was rinsed with the washing buffer (repeated three times), resulting in purified DNA intercalator-modified AuNS. The 327 μl, 201 μl and 63 μl of 10 nM AuNS solution in 10 mM Tris-HCl buffer (pH 8) were mixed with 10 μl of 1 nM pBR322, pUC18 (both from *Escherichia coli* RR1, obtained from Sigma-Aldrich) and open-circular pRQ7 DNA (*Thermotoga* sp. RQ7 plasmid pRQ7, obtained from Bioneer), respectively, and incubated for 24 h with gentle shaking followed by centrifugal filtration to remove free AuNS. The AuNSs conjugated with plasmids were used immediately after the intercalation process.

### Setting of nanoplasmonic characterization

First Contact cleaning polymer (Photonic Cleaning Technologies) was applied onto microscope slides (22 × 40 × 0.1 mm; Warner Instruments) and peeled off immediately after a cure for 15 min. Then the slide was cleaned overnight using freshly prepared aqua regia solution, rinsed with ultra-pure water, dipped in 5% (v/v) 3-aminopropyltriethoxysilane in absolute ethanol for 15 min and sonicated in ultra-pure water for 5 min (repeated three times). Three microlitres of diluted AuNS solution (OD ∼0.05) was dropped onto the silanised slide. After incubation for 1 min at room temperature, the slide was washed with ultra-pure water and ethanol in a biological hood to minimize contaminations with airborne debris, and finally blow-dried with nitrogen gas. The Rayleigh scattering properties of each AuNS were detected on the slide installed onto the stage controller of the sensing system (Supplementary Fig. 4). To analyse the growth dynamics of AuNS, the slide was mounted onto a closed-bath imaging chamber with microchannels (RC-30, Warner Instruments). The chamber was assembled onto the stage controller and connected with a flow device enabling fast turbulent solution mixing and a flow-rate control platform (PHD 2,000, Harvard Apparatus). Then, NH_2_OH solution and HAuCl_4_ solution flowed into the chamber at a flow rate of 10 μl min^−1^. Using a dark-field microscope (Eclipse TE2000-U, Nikon), light-scattering objects are brightly illuminated against the background in the image. At a resonance wavelength, the optical size of individual nanoparticles is strongly enlarged by Rayleigh light scattering, allowing the recognition of a single nanoparticle with the naked eye using a field of view of the dark-field microscope. The field of view of the microscope was taken by a colour camera (D50, Nikon). A beam splitter at the output port of the microscope and a long pass filter were placed before a charge-coupled device (CCD) camera (PIXIS: 400B, Princeton Instruments). Focused images of the chamber were projected onto a −70 °C CCD with a frame integration time of 100 ms. For spectral measurements by Rayleigh light scattering spectrograph (Microspec 2300i, Princeton Instruments), the focused image of one target particle was projected onto the end of a 600-mm-diameter optical fibre, and the spectra in a range of 300–900 nm were subsequently recorded with acquisition time of 5 s. To reduce inter-particle resonance coupling effects^69^, only individual nanoparticles with a particle-to-particle spacing larger than ∼5 times the particle diameter were selected for detections.

### Characterization of AuNCs

UV–*vis* absorption spectra of AuNCs in aqueous solution were obtained using an ultraviolet–visible–near-infrared spectrophotometer (Shimadzu UV-3600) at a wavelength range of 300–1,100 nm. Surface-enhanced Raman scattering of AuNCs conjugated with malachite green isothiocyanate^70^ was measured using a Renishaw 2,000 Raman microscope system. A Spectra Physics He-Ne laser (Research Electro-Optics) operating at *λ*=633 nm was used as the excitation source with a laser power of ∼12 mW. Cold field emission scanning electron microscope images were taken at an accelerating voltage of 15 kV (Hitachi S-4300) with silicon chip specimen supports (Ted Pella). Atomic force microscopy (AFM) was conducted at room temperature using a multimode scanning probe microscope with a Nanoscope 3D controller (Digital Instruments/Veeco Probes). AFM topographical analysis was performed using Ultrasharp SiN AFM tips (MikroMasch) in tapping mode at their resonant frequency, and the images were analysed with WsXM scanning probe image processor. TEM and HR-TEM images were obtained under a Hitachi HD2300 transmission electron microscope in z-contrast and SE mode at an accelerating voltage of 300 kV. The samples for TEM were prepared using an EMS staining plate (electron microscopy sciences) and 400-mesh copper TEM grids with carbon film (Ted Pella). Thiol-functionalized smart grids (Structure Probe) were used for the investigation of plasmid DNA-conjugated AuNS and the growth processes. FFT patterns of HR-TEM images were analyse by Digital Micrograph software (Gatan Inc.) to confirm the crystalline structures and the growth orientations. Energy dispersive X-ray (EDX) spectroscopy with HR-TEM was performed at an accelerating voltage of 300 kV to identify elements of the nanocrystals.

### Data availability

The authors declare that the data supporting the findings of this study are available within the article and its Supplementary Information.

## Additional information

**How to cite this article:** Ma, X. *et al.* Gold nanocrystals with DNA-directed morphologies. *Nat. Commun.* 7:12873 doi: 10.1038/ncomms12873 (2016).

## Supplementary Material

Supplementary Information Supplementary Figures 1-16, Supplementary Tables 1-2, Supplementary Notes 1-2 and Supplementary References
