# Supplementary material for: Gold nanocrystals with DNA-directed morphologies
Source: Nat Commun. 2016 Sep 16;7:12873. doi: 10.1038/ncomms12873 (PMC5028415; doi:10.1038/ncomms12873)
Supplement: Supplementary Information — Supplementary Figures 1-16, Supplementary Tables 1-2, Supplementary Notes 1-2 and Supplementary References [file ncomms12873-s1.pdf]

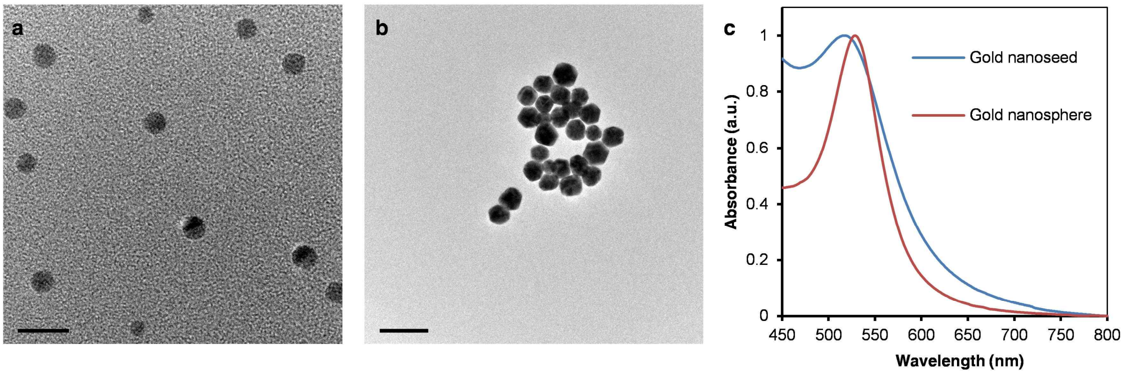

Supplementary Figure 1 | Characteristics of gold nanoseeds (AuNSs) and control experiment of crystallisation without DNA. **a**, TEM image of AuNSs purchased from British BioCell International (~5 nm in diameter). Scale bar, 10 nm. **b**, TEM image of gold nanospheres resulted in the control experiment (~36.3 nm in diameter). Instead of AuNS-L1, bare AuNSs in the absence of DNA was used in the control experiment under the same conditions. The control experiment confirmed the regulating effect of DNA during the Au crystallisation. Scale bar, 100 nm. **c**, Normalised UV-*vis* spectra of the AuNSs and the gold nanospheres.

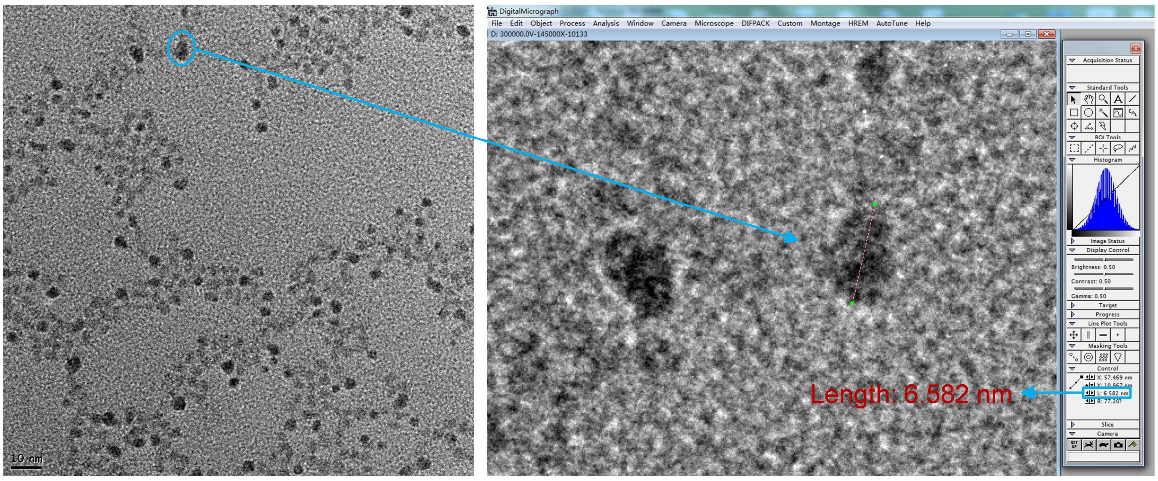

Supplementary Figure 2 | Measurement of AuNC dimensions. The dimension data were obtained by measuring the dimensions of 50 AuNCs or more in one EM image (*left*) using DigitalMicrographTM software (Gatan Inc.; *right*).

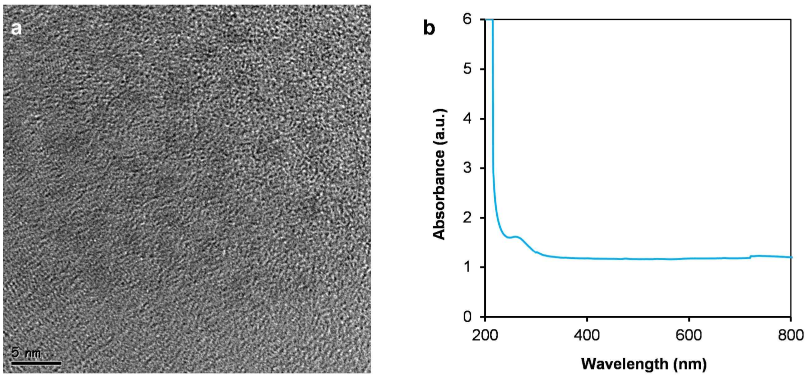

Supplementary Figure 3 | Control experiment of crystallisation without AuNS. a, A typical TEM image of the product after mixing DNA, the reductant and gold precursor. Without AuNSs, the reaction system did not generate AuNCs, indicating the indispensable role of AuNSs in initiating Au crystallisation along DNA. The result was further confirmed by UV-*vis* spectroscopy as shown in **b**.

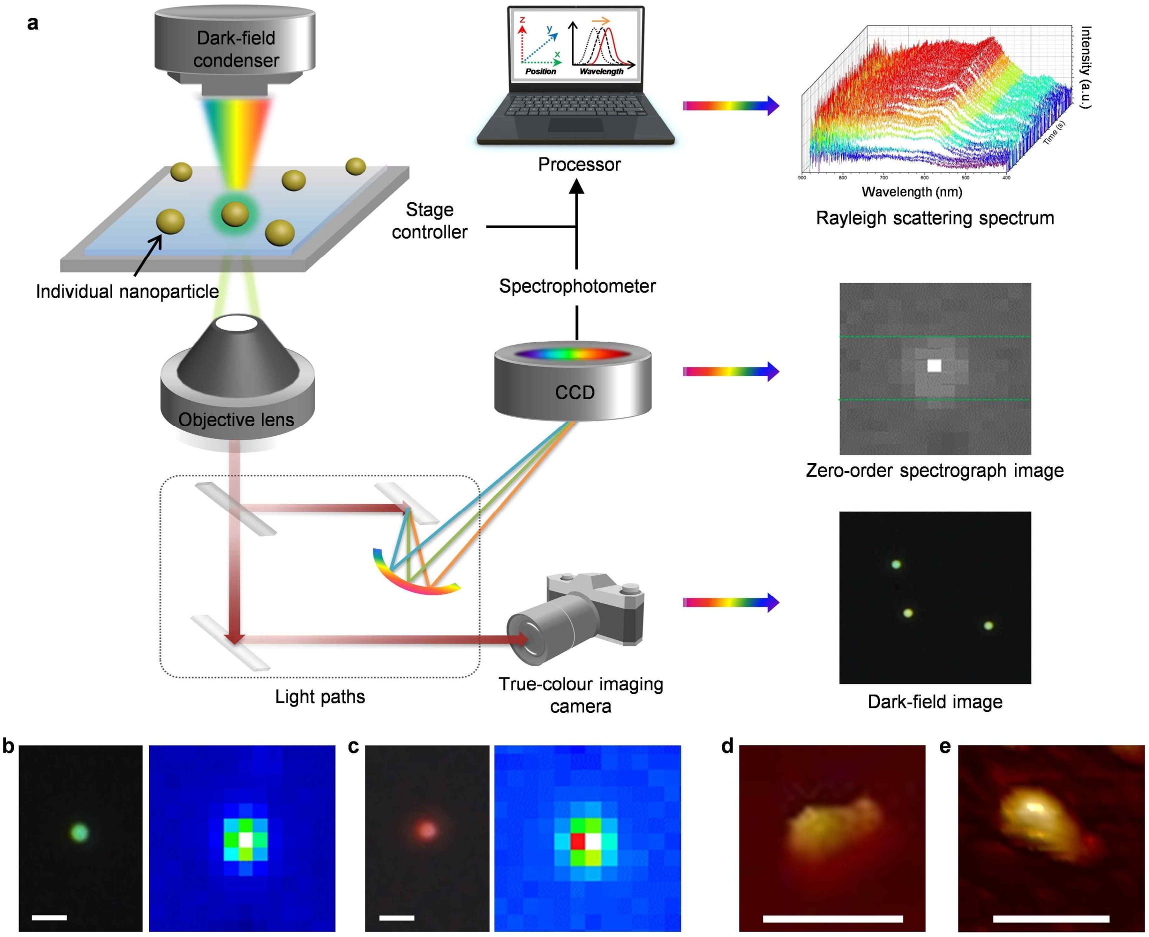

Supplementary Figure 4 | *In situ* investigations of the DNA-directed crystallisation of Au in a sequence-independent manner. a, Detailed configuration of the nanoplasmonic sensing system and sample data obtained from the system. The profiles of a single nanoparticle are obtained using the integrated system consisting of a 100-W tungsten lamp, a dark-field condenser, a microfluidic device, a high-precision stage controller, an oil-immersion objective, a colour camera, a Rayleigh light scattering spectrograph, a highly sensitive CCD camera, and a data analysis unit. The *in situ* images were taken by dark-field microscopy (dark background) and CCD camera (blue background) for a AuNC synthesised without DNA (**b**) and a AuNC synthesised with single dsDNA (**c**). Scale bar, 1 μm for all. **d**, AFM image of a AuNC directed by 30-bp linear dsDNA with the sequence shown in Supplementary Table 2. AuNC with identical morphology was directed by using dsDNA with poly(A-T) sequence (**e**). The poly(A-T) dsDNA was prepared using polyA30 and polyT30 ssDNA. Both scale bars, 100 nm.

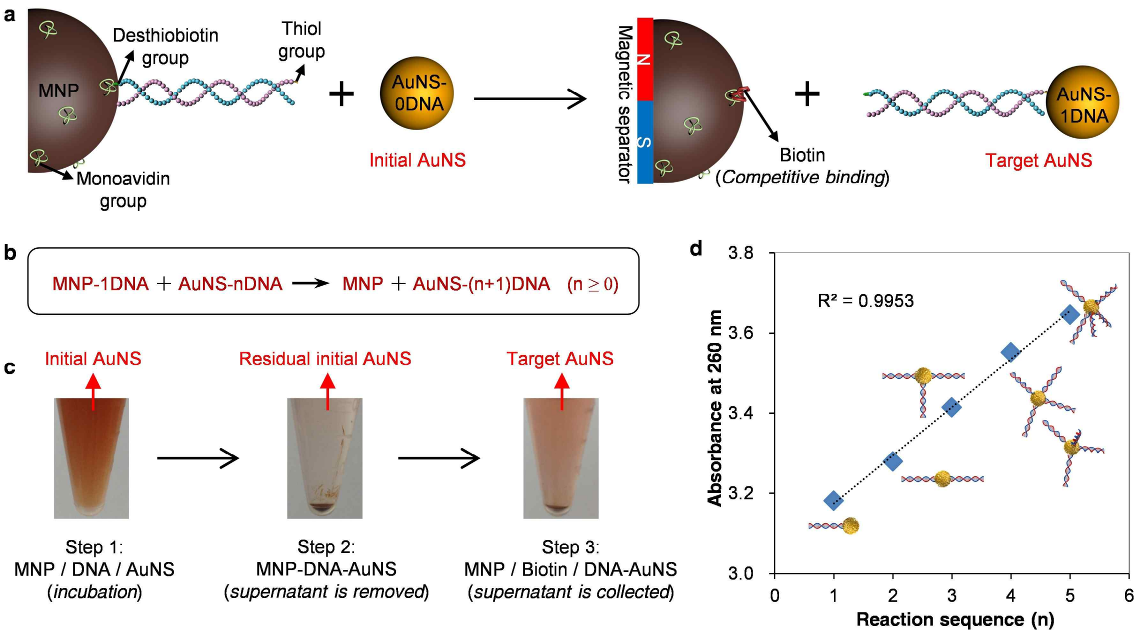

Supplementary Figure 5 | Conjugation of AuNSs with linear DNA. a, Schematic illustration of the functional groups for "one-after-another" conjugation of DNA onto a AuNS. One end of the DNA modified with desthiobiotin group was conjugated with the monoavidin group on the MNP surface, while the other end with thiol group bound onto AuNS. The AuNS-DNA was separated from the MNP surface by adding biotin, which has a stronger binding affinity with monoavidin than desthiobiotin. **b**, Equation showing the one-after-another approach. "AuNS-nDNA" is the initial nanoseed for "AuNS-(n+1)DNA". **c**, Photos showing each step in the conjugation process. The *initial AuNS* was used in excess to maximise the yield of MNP-DNA-AuNS whereas the *residual initial AuNS* can be recycled for other usage after the mild separation process. The *residual initial AuNS* was rinsed-off to improve the monodispersity of *target AuNS*. **d**, Statistic analysis of the DNA binding profile by UV-*vis* spectroscopy. Given a benchmark number of AuNSs (by normalising the absorbance at 518 nm to be 1), the number of DNA (by estimating the maximum absorption intensity at 260 nm) shows a good linear relationship with the reaction sequence (n).

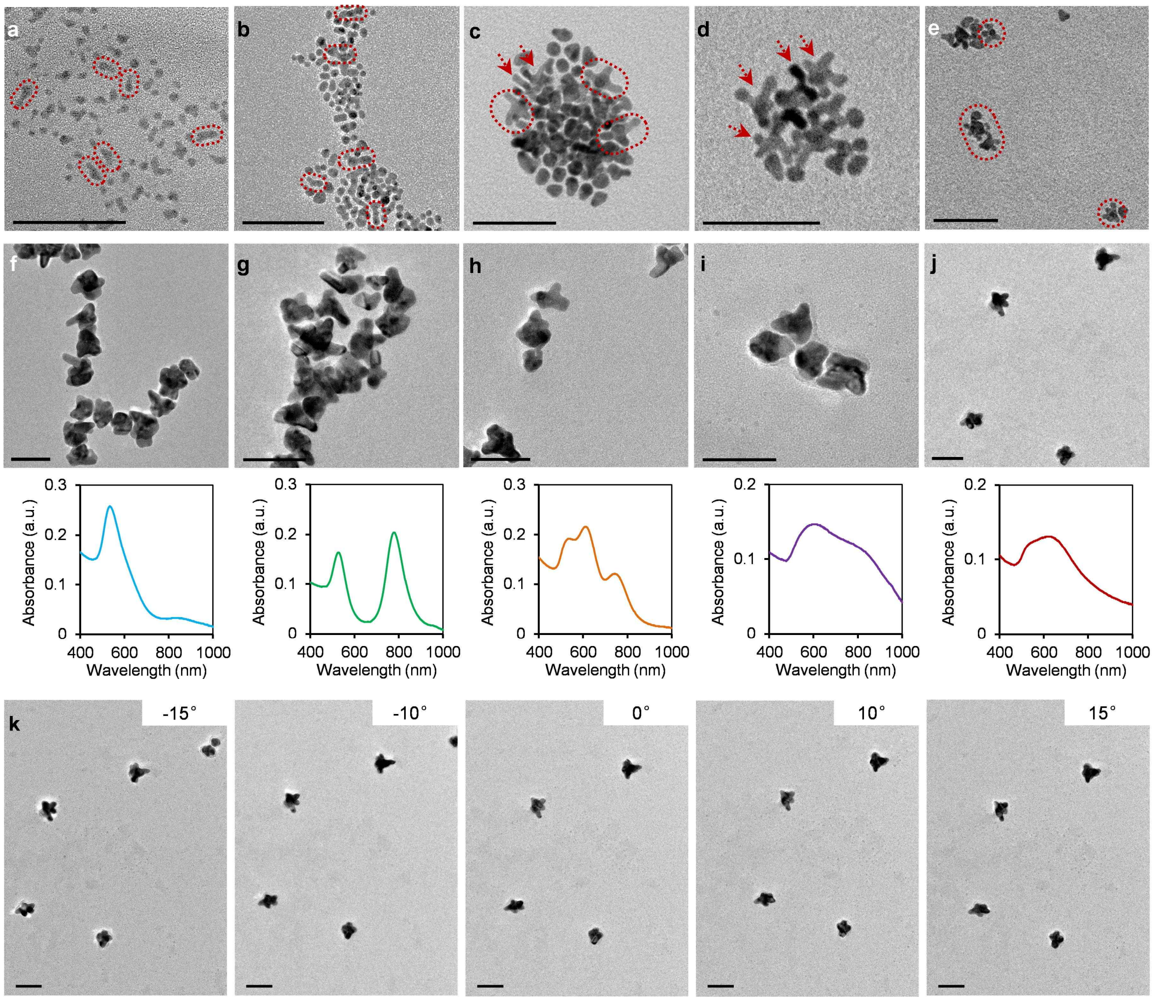

Supplementary Figure 6 | Linear dsDNA-directed crystallisation of star-shaped AuNCs. a–e, TEM images of AuNSs directed by dsDNA in varying numbers of 1 to 5, respectively, synthesised with 3.8 μL of gold precursor. **f–j**, AuNSs directed by dsDNA in varying numbers of 1 to 5, respectively, synthesised with 57 μL of gold precursor. The corresponding UV-*vis* absorption spectra are shown below the TEM images. **k**, The AuNCs directed by AuNS-L5 was investigated by a tilt series of TEM using a 5° tilt interval from –15° to +15°. All scale bars: 50 nm.

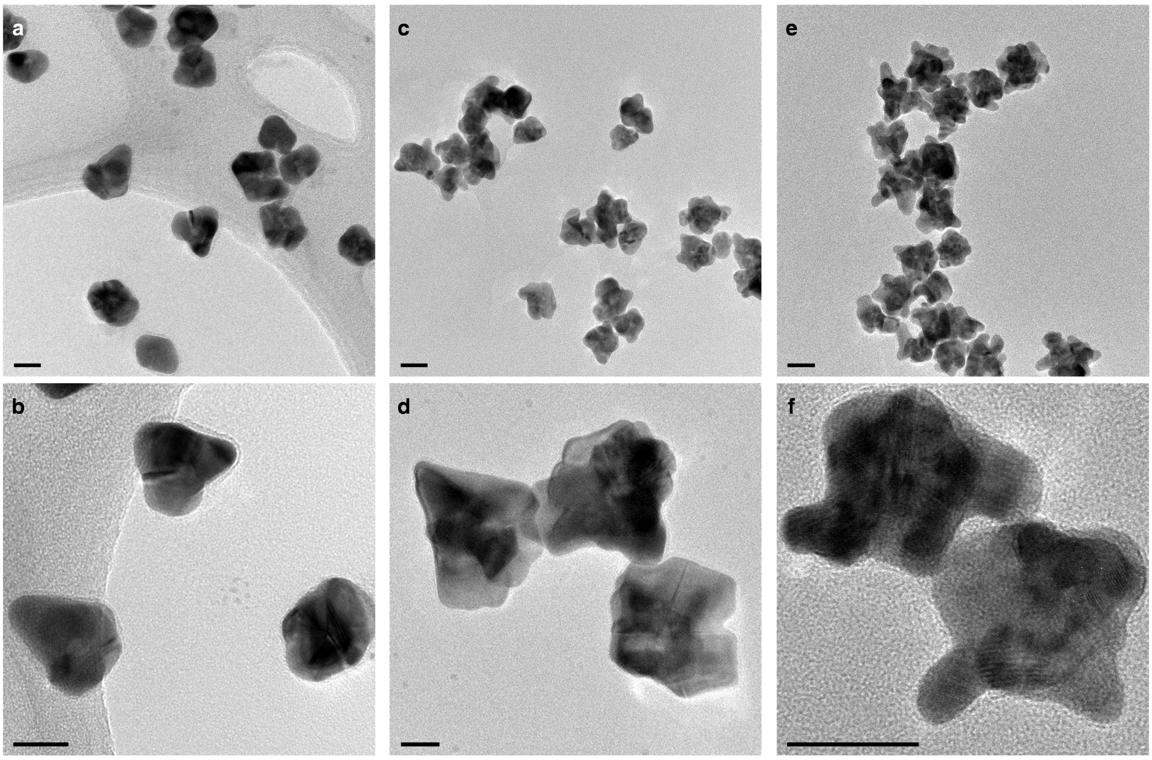

Supplementary Figure 7 | ssDNA-directed crystallisation of AuNCs. TEM images of AuNCs whose synthesis was directed by 1 (**a** and **b**), 3 (**c** and **d**), 5 (**e** and **f**) ssDNA strand, per AuNS. All scale bars: 20 nm.

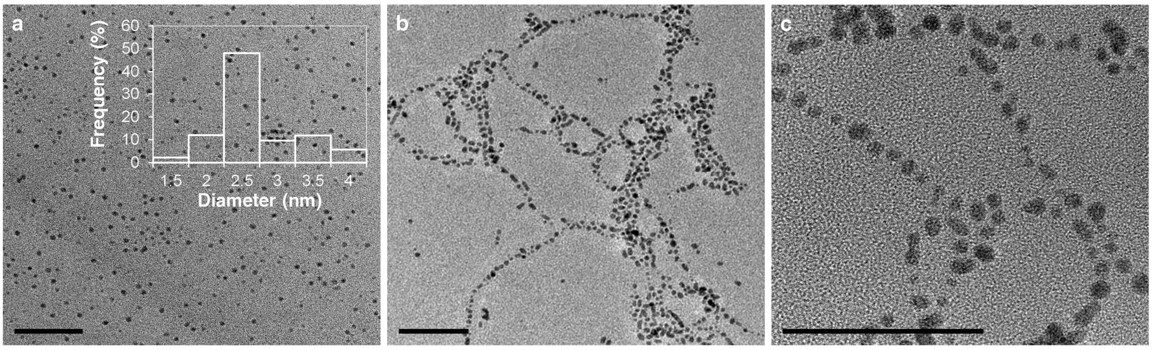

Supplementary Figure 8 | Intercalation of AuNSs in plasmid DNA. a, TEM image of AuNS before the intercalation. Inset: size distribution. **B** and **c**, TEM images of AuNS intercalated in pUC18 plasmid DNA. All scale bars: 50 nm.

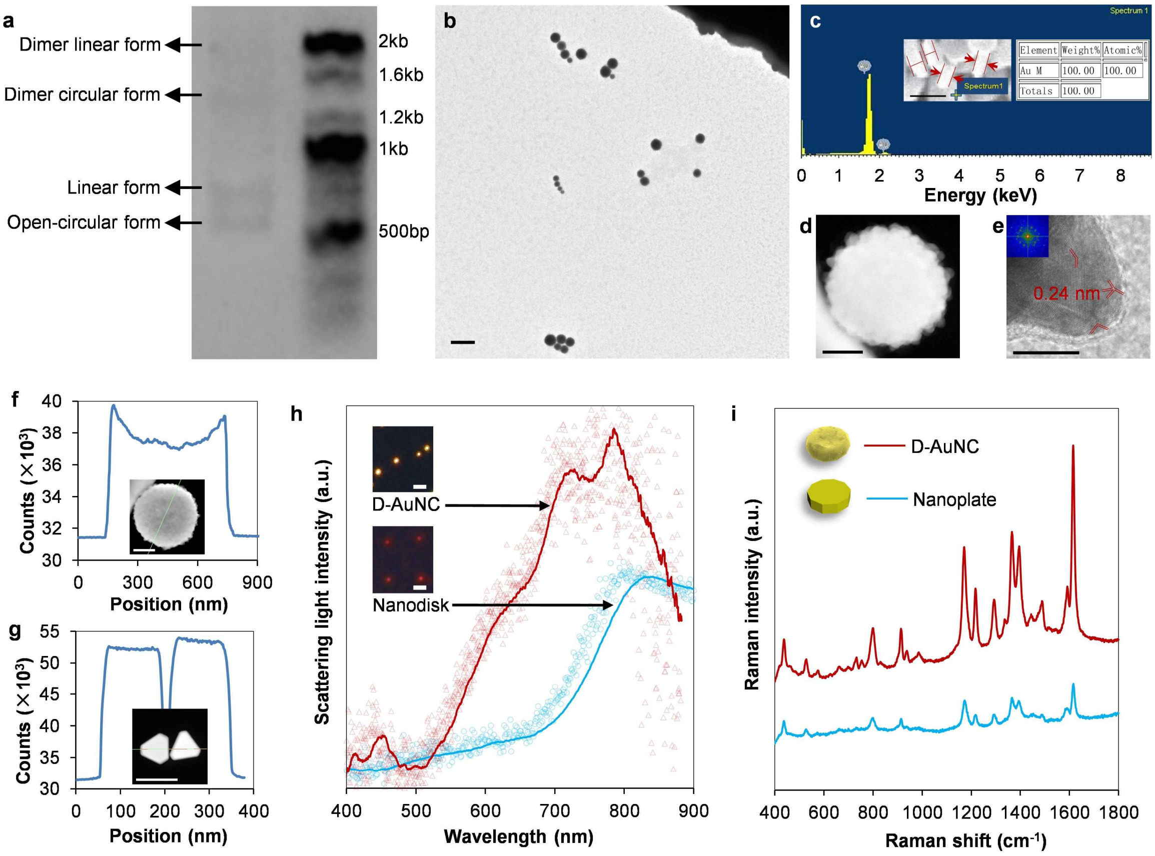

Supplementary Figure 9 | Open-circular dsDNA-directed crystallisation of D-AuNCs. **a**, Characterisation of the open-circular DNA by electrophoresis. **b**, TEM image of the D-AuNCs. Scale bar, 500 nm. **c**, SEM image and EDX analysis of the D-AuNC. Scale bar, 250 nm. **d**, HAADF-STEM image of the D-AuNC. Scale bar, 100 nm. **e**, HR-TEM image of tip on D-AuNC's edge. Inset: the corresponding FFT image. The lattice fringes at ~0.24 nm spacing distance indicate that the crystal is enclosed by stable {111} planes. Scale bar: 10 nm. **f**, Scanning line profile of the surface, with a control test using flat-surfaced nanoprisms (**g**). Both scale bars: 200 nm. **h**, Rayleigh light scattering spectra and dark-field images of D-AuNC and nanodisk fabricated by conventional lithography. Both scale bars: 10 μm. **i**, SERS spectra of malachite green isothiocyanate enhanced by D-AuNCs and hexagonal nanoplates synthesised by wet-etching methods. D-AuNC shows an enhancement factor 6 times higher than the nanoplate.

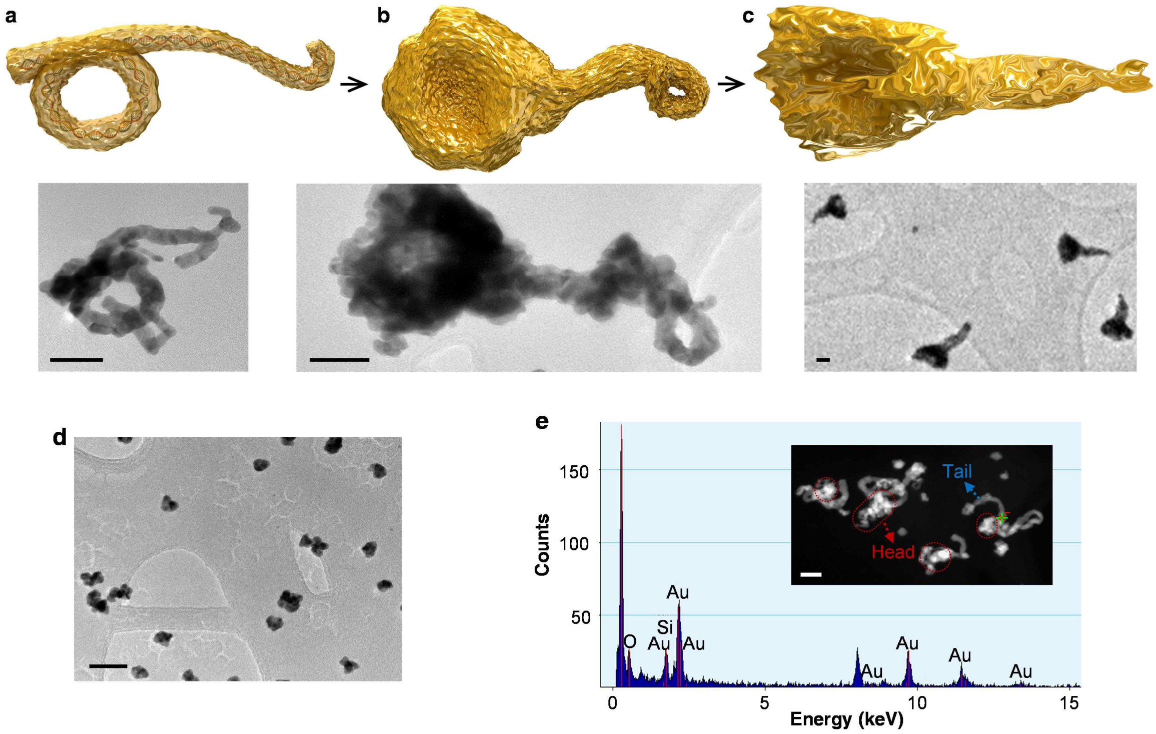

Supplementary Figure 10 | Plasmid (pUC18)-directed growth process of gold nano-jellyfish. a–c, Schematic diagrams and TEM images of the intermediate nanostructures during growth. The large supercoiled dsDNA strand unwound and transformed into a structure with a circular-coil and a long-tail during reactions as shown in **a**. The structure further directed the gold crystallisation and formed the head and tentacle of jellyfish-like structure (**b** and **c**). **d**, The structural transformation process dissociated some AuNSs from the plasmid which finally grew into small polyhedral nanospheres as shown in the TEM image. **e**, EDX spectrum taken from the nanostructure after structural transformation of plasmid. Inset: STEM image of the nanostructure. All scale bars: 50 nm.

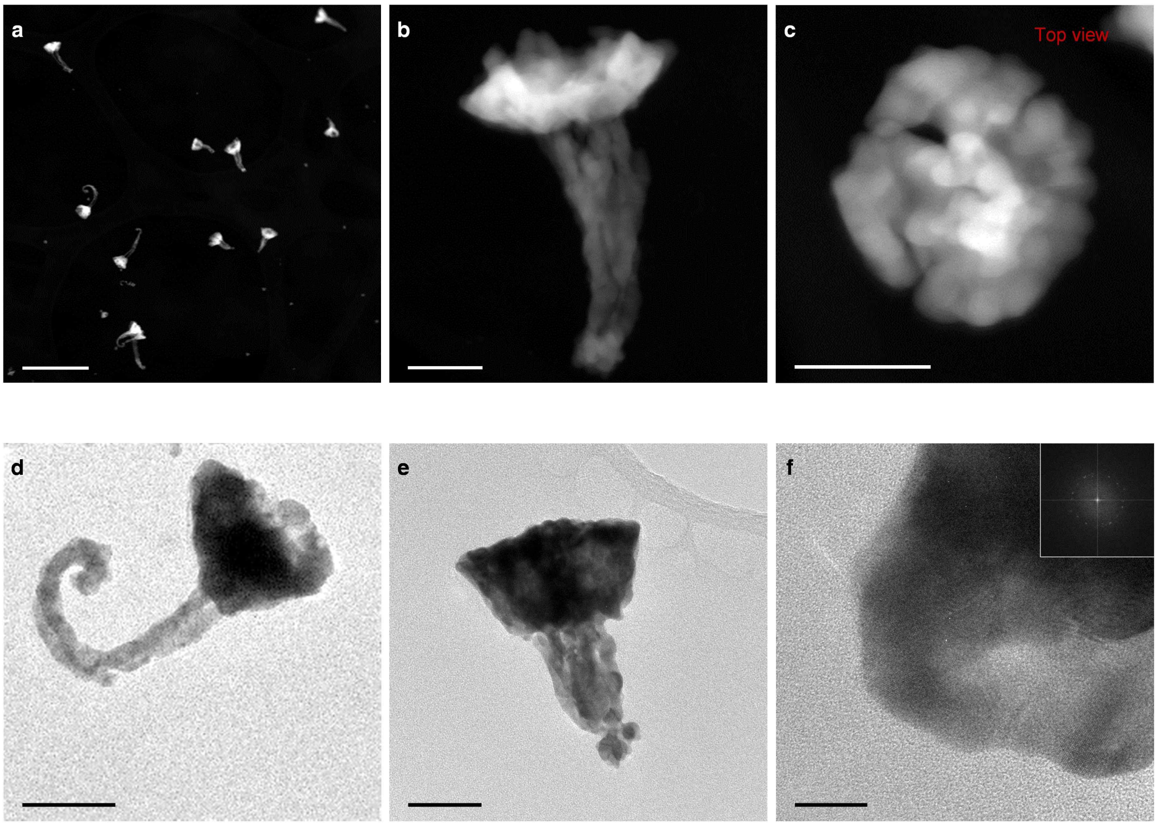

Supplementary Figure 11 | Plasmid pUC18-directed crystallisation of jellyfish-like AuNCs. **a**, STEM image of the jellyfish-like AuNCs. Scale bar, 500 nm. **b** and **c**, HAADF-STEM images of the AuNCs. Both scale bars, 50 nm. **d** and **e**, TEM images of the AuNCs. Both scale bars, 50 nm. **f**, HR-TEM image of the head of a “jellyfish” AuNC. Inset: the corresponding FFT image. Scale bar, 10 nm.

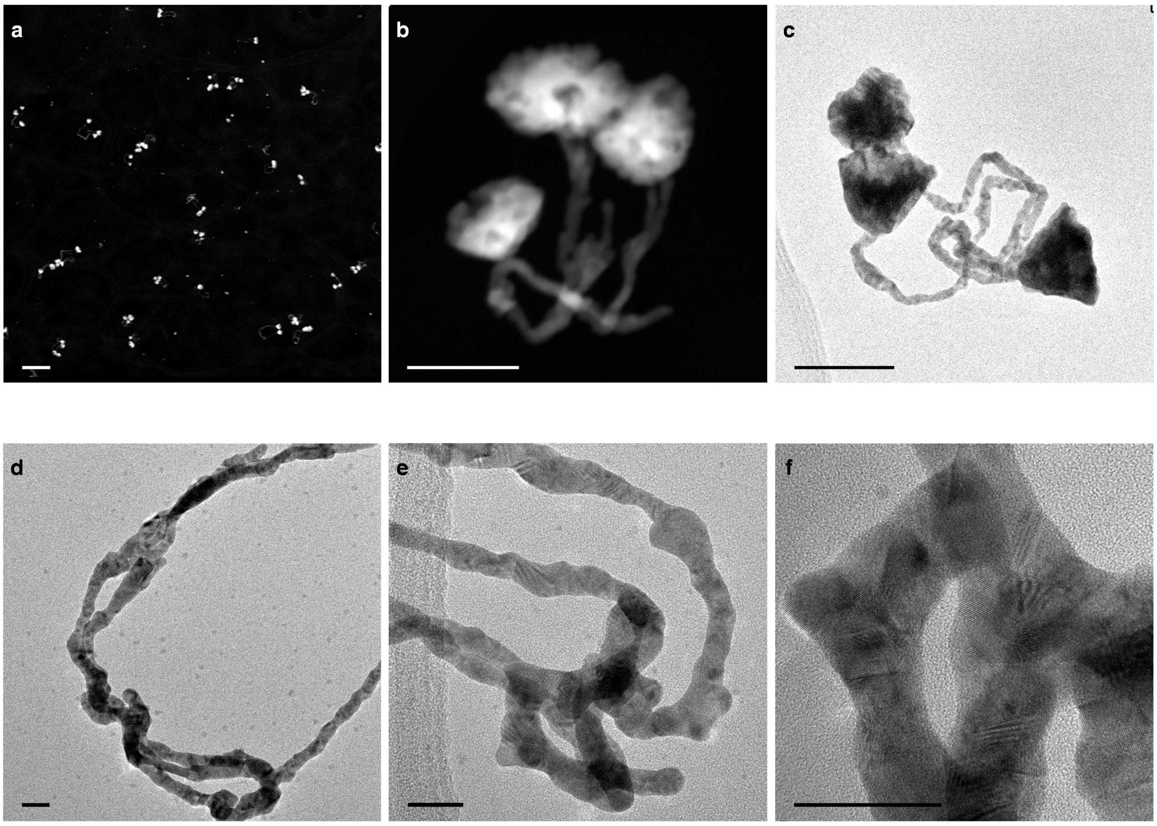

Supplementary Figure 12 | Plasmid pBR322-directed crystallisation of the flower-like AuNCs. a, STEM image of the flower-like AuNCs. Scale bar, 500 nm. **b**, HAADF-STEM image and **c**, TEM image of the AuNC. Both scale bars, 100 nm. **d–f**, HR-TEM images of the flexibly extended stalks of a "flower-like" AuNC. All scale bars, 20 nm.

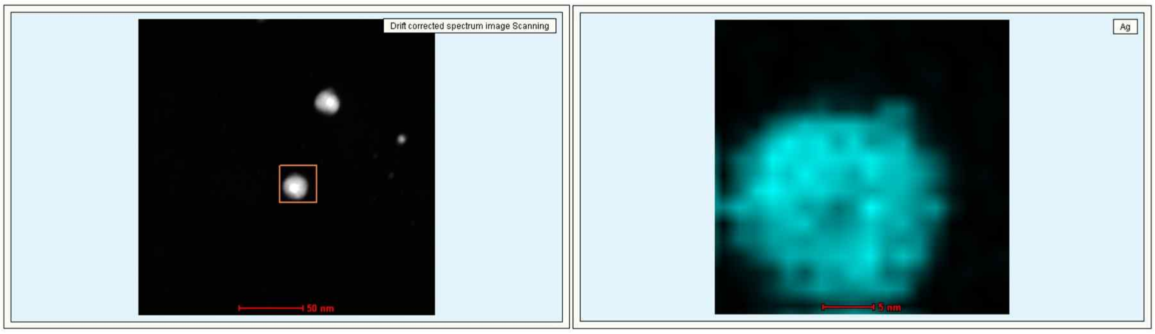

Supplementary Figure 13 | Control experiment of crystallisation using positively charged metal precursor (Ag^+). $AgNO_3$ was used instead of $HAuCl_4$ in the AuNS-L1 reaction system. The growth of Ag did not follow the direction of dsDNA but resulted in rough-surfaced spherical nanocrystals as shown in the STEM image (*left*) with the corresponding EDS element map (*right*).

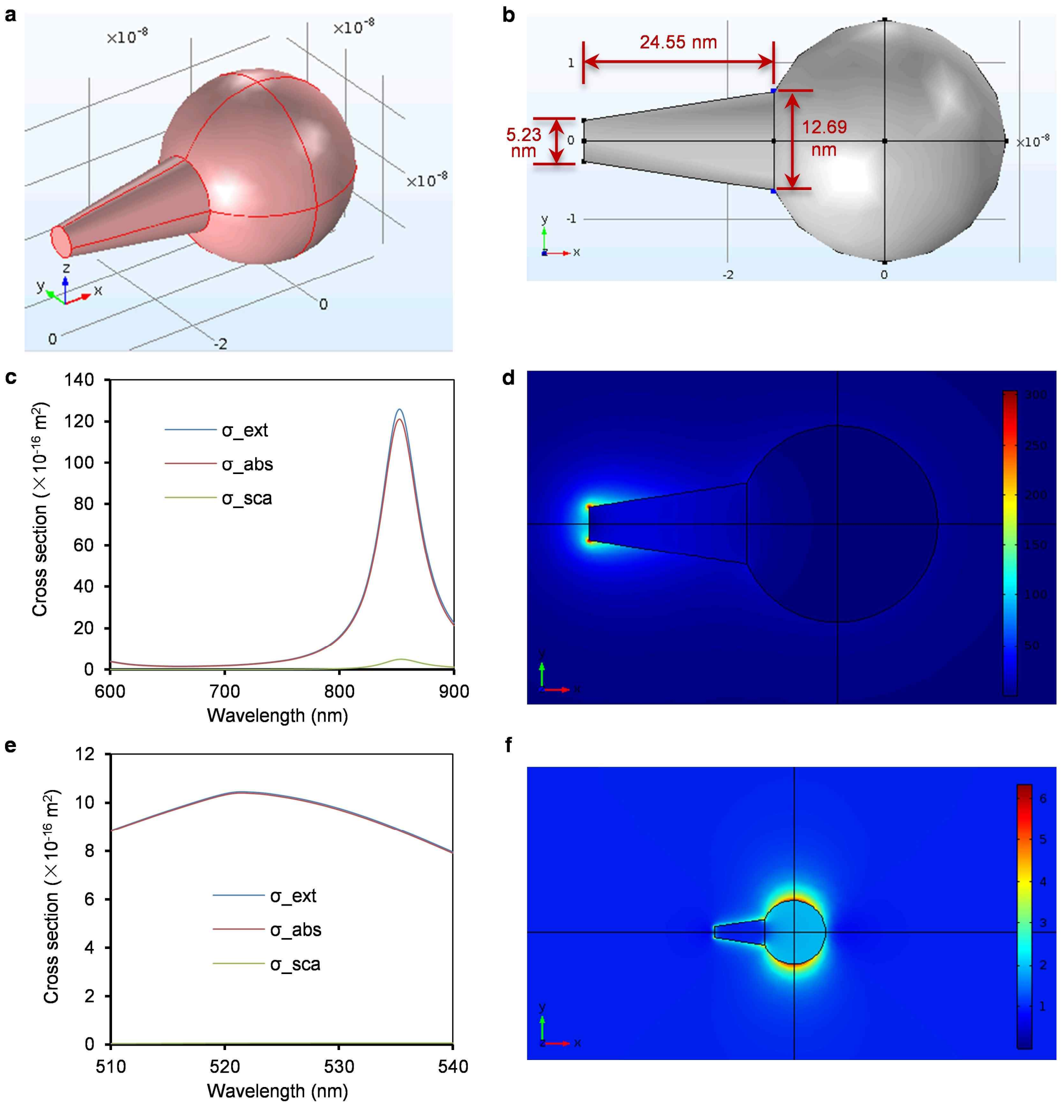

Supplementary Figure 14 | Numerical simulation of optical properties of AuNCs directed by AuNS-L1. a, Perspective view of the AuNC model. **b**, AuNC model with dimensions. **c**, Calculated extinction, absorption and scattering cross-section for light polarized along the branch. **d**, Electric field pattern of the plasmon resonance at 850 nm. **e**, Calculated extinction, absorption and scattering cross-section for light polarized perpendicular to the branch. **f**, Electric field pattern of the plasmon resonance at 523 nm.

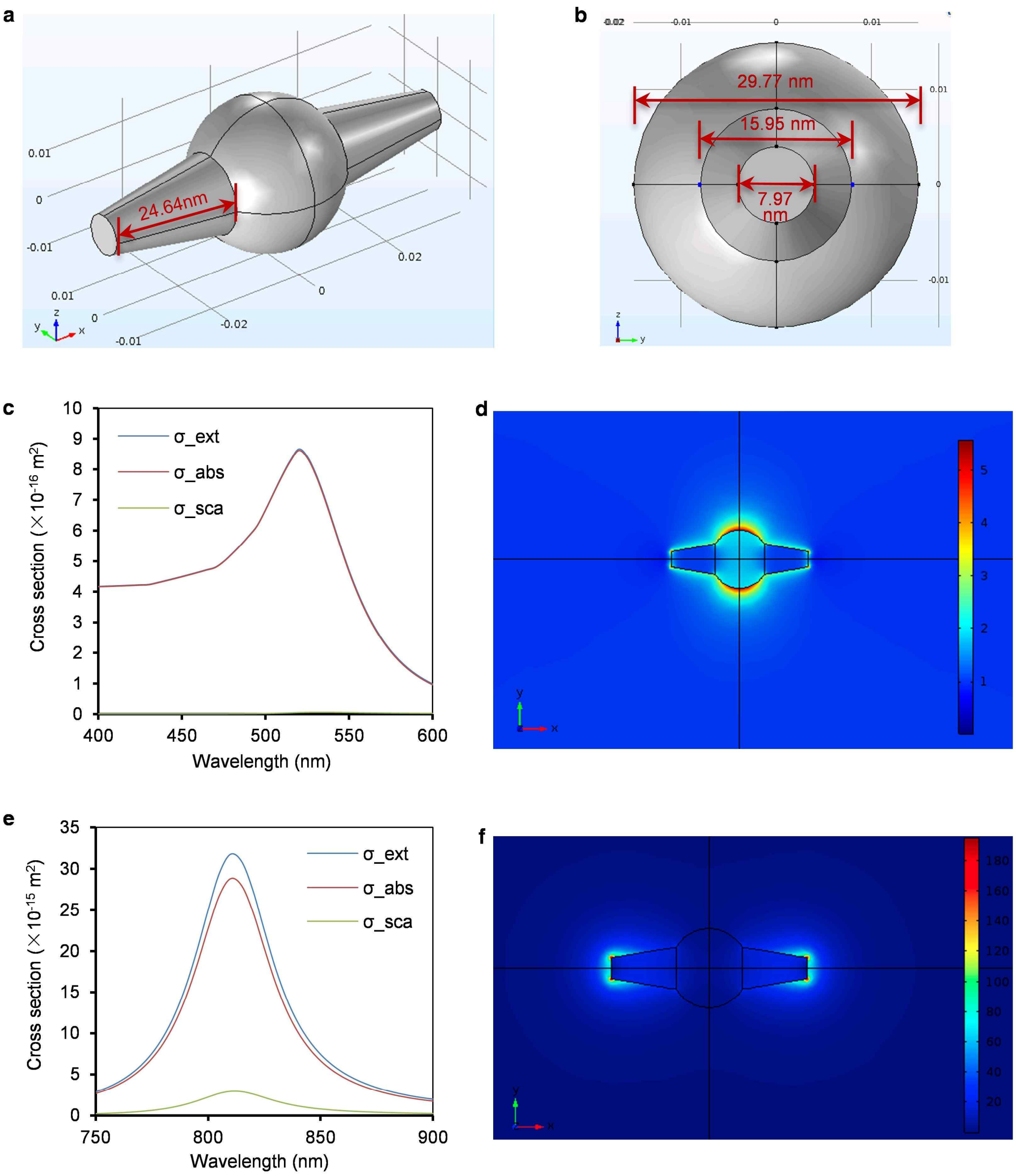

Supplementary Figure 15 | Numerical simulation of optical properties of AuNCs directed by AuNS-L2. a, Perspective view of the AuNC model. **b**, Side view of the AuNC model. **c**, Calculated extinction, absorption and scattering cross-section for light polarized perpendicular to the branches. **d**, Electric field pattern of the plasmon resonance at 520 nm. **e**, Calculated extinction, absorption and scattering cross-section for light polarized along the branches and **f**, Electric field pattern of the plasmon resonance at 810 nm.

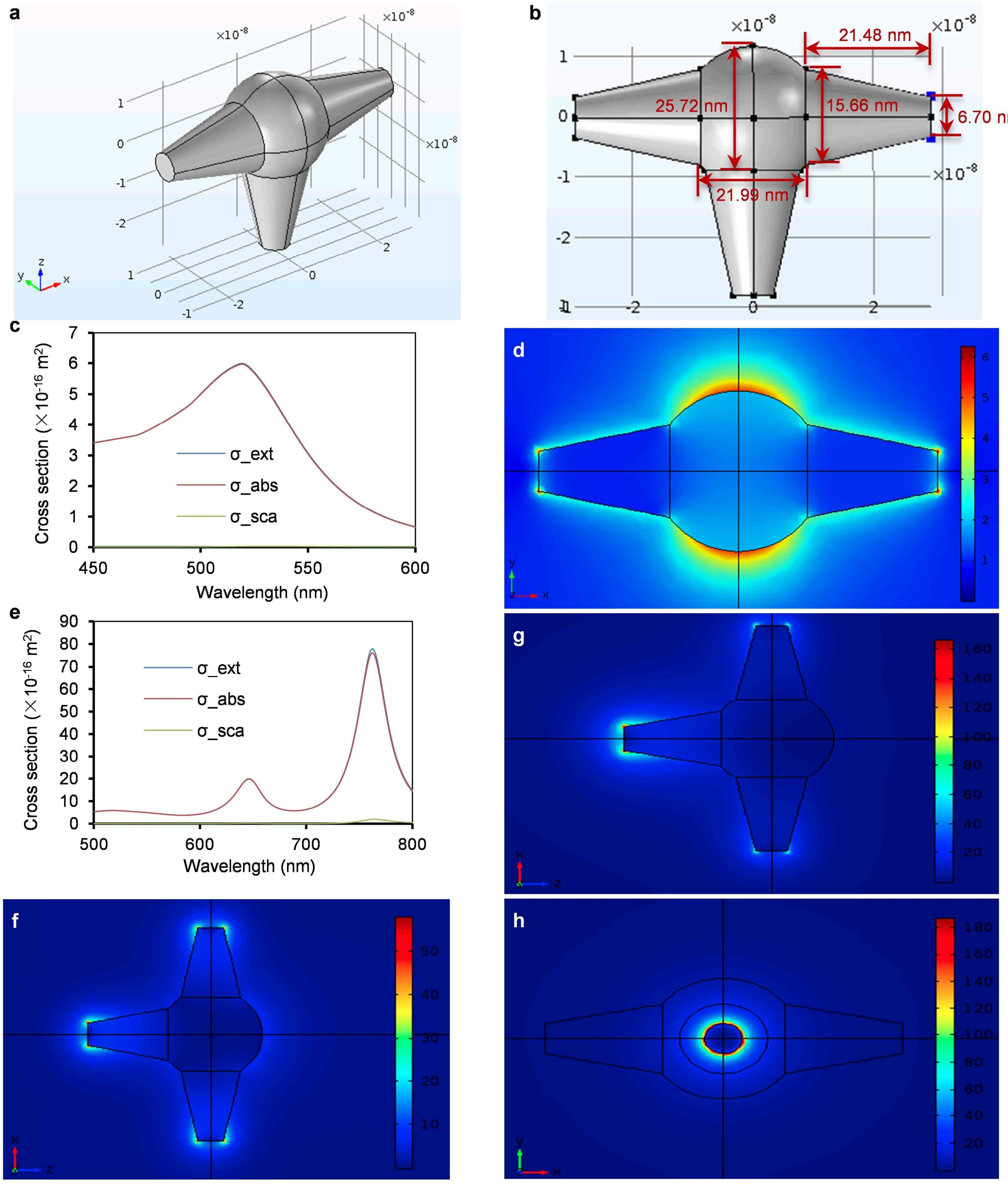

Supplementary Figure 16 | Numerical simulation of optical properties of AuNCs directed by AuNS-L3. a, Perspective view of the AuNC model. **b**, Side view of the AuNC model with dimensions. **c**, Calculated extinction, absorption and scattering cross-section for light polarized along the branches. **d**, Electric field pattern of the plasmon resonance at 520 nm. **e**, Calculated extinction, absorption and scattering cross-section for light polarized perpendicular to the branches. **f**, Electric field pattern of the plasmon resonance at 645 nm. **g** and **h**, Electric field pattern of the plasmon resonance at 762 nm.

Supplementary Table 1 | Statistic analysis of linear DNA-directed AuNCs.

AuNCs	Total count	Yield in desired morphology	Core diameter[1] (nm)	Branch length[1] (nm)	Branch diameter[1] (nm)		
					At the front	At a half length	At the base
AuNS-L1	92	66.3%	30.88 ± 4.5	24.52 ± 3.1	5.23 ± 0.6	10.93 ± 1.2	12.80 ± 1.9
AuNS-L2	179	55.9%	29.77 ± 3.8	24.35 ± 4.4	7.97 ± 1.4	12.58 ± 2.2	15.90 ± 0.6
AuNS-L3	125	53.6%	23.54 ± 2.8	21.82 ± 3.5	6.70 ± 1.3	13.63 ± 1.6	15.94 ± 2.1
AuNS-L4	335	50.7%	23.46 ± 4.4	19.01 ± 3.3	5.62 ± 1.0	11.79 ± 1.1	13.93 ± 2.3
AuNS-L5	72	56.9%	20.76 ± 2.4	15.97 ± 2.8	3.26 ± 0.7	7.53 ± 1.5	10.64 ± 2.1

[1] Dimension diagram:

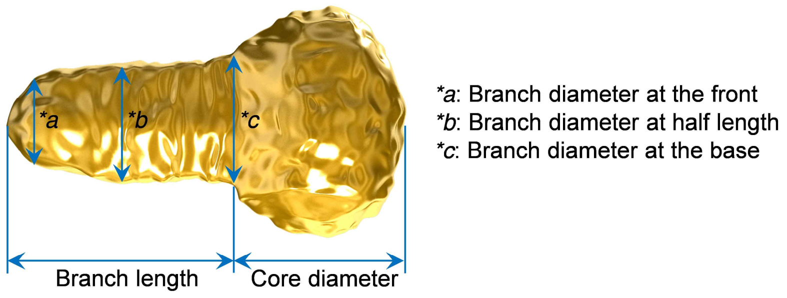

Supplementary Table 2 | DNA sequence information.

Assigned name	Modification	Sequence (5′—3′)
30-bp linear dsDNA1	5′-desthiobiotin	GAGCATACATAGGGTTTCTCTTGGTTTCTT
30-bp linear dsDNA2	5′-thiol	AAGAAACCAAGAGAAACCCTATGTATGCTC
30-bp linear ssDNA	5′-desthiobiotin<br>3′-thiol	GAGCATACATAGGGTTTCTCTTGGTTTCTT
polyA30	5′-desthiobiotin	AAAAAAAAAAAAAAAAAAAAAAAAAAAAAA
polyT30	5′-thiol	TTTTTTTTTTTTTTTTTTTTTTTTTTTTTT

Supplementary Note 1 | Comparison of DNA-mediated approaches in nanomaterial synthesis and advantages of DNA-directed nanostructures.

Major differences between the previously reported DNA-templated metallisation and the current DNA-directed crystallisation are in synthesis procedures, mechanisms, and final products.

DNA-templated metallisation: (1) Anionic DNA or DNA-assembly absorbs onto a solid substrate in its pattern, (2) cationic metal ions or their complexes bind onto DNA by attractive electrostatic forces or π stacking, (3) the bound metal ions or complexes are reduced by an exogenous reductant and concomitantly attach to the DNA as small metal clusters, and (4) the metal clusters serve as nucleation sites for forming either sequential necklaces or continuous bulges with a poorly controlled structural precision. "Electroless plating" might be a more precise terminology in chemistry for this process than crystallisation.

DNA-directed crystallisation: (1) The synthesis is carried out by Au crystallisation in a colloidal solution, (2) anionic $AuCl_4^-$ gets reduction without directly reacting with DNA, (3) the reduction continues directionally along DNA, and (4) finally a crystalline body in nanoscale is formed around DNA, rather than aggregative granular objects.

It was previously shown that the synthesis of colloidal NCs in controlled shapes was limited only to symmetric ones with identical surface facets (polyhedral structures; control resolution in a scale of 10 nm)[1,2]. The synthesis of irregularly shaped nanocrystals was empirical rather than scientific, without principles to follow[3]. The controllability is hampered by lacking organic molecules that are able to specifically coat targeted crystal facets and thereby produce nanomaterials with desired structures. A strategy used in a recent study to cast asymmetric shapes using pre-defined molds was quite complex with a low casting yield[4]. We synthesised asymmetric nanocrystals in a desired direction (e.g. a branched form of nanocrystals; named "nanostars") with a relatively high morphological yield. Nanostars generally exhibit stronger local electric field than polyhedral and irregularly shaped nanocrystals because the nano-branches provide natural focusing of

electromagnetic field. This is a phenomenon similar to the fact that nanorods typically exhibit higher local electromagnetic field than nanospheres. Compared to nanorods, nanostars offer more branches that can support optical hot spots. For biosensing or diagnostics applications, more hot spots per nanocrystal naturally lead to higher sensitivity. Compared to conventionally synthesised nanostars with uncontrollable branches produced using surfactants[5-13], the DNA-directed nanostars developed in the current study offer a significant advantage to facilely tune the local electromagnetic field by their DNA-directed branches (e.g., UV-*vis* spectra shown in this study).

Supplementary Note 2 | Numerical simulations of optical properties of star-shaped AuNCs.

We further conducted optical simulations using the commercial software COMSOL to understand the plasmon behaviour of the star-shaped AuNCs with one branch (AuNC-1), two branches (AuNC-2) and three branches (AuNC-3). In the modelling, the nanostar geometry is simplified in such a way that the main characteristics important for the optical properties are captured while the unimportant details (e.g. surface roughness, slight curvatures of the branches, etc.) are ignored. More specifically, the AuNCs are modelled as a spherical core with different number of branches attached to it. The branches ware modelled as tapered cylinders. This modelling study is focused on identifying the origins of the various extinction peaks observed in experiments (Supplementary Fig. 6). It does not attempt to precisely reproduce the spectra, as the details of the spectra should depend on the relative concentrations of all nanoparticles in the solution. Instead, the expected extinction peak positions and the nature of the plasmon resonance responsible for each peak are determined.

AuNC-1 is modelled as a spherical core with one tapered cylindrical branch (Supplementary Fig. 14a,b). The extinction, scattering, and absorption cross-section for various incident light polarizations and nanostar orientations are calculated. The AuNC-1 is found to exhibit two plasmon resonances at 850 nm and 523 nm (Supplementary Fig. 14c-f). The former corresponds to the plasmon resonance concentrated at the tip of the branch (Supplementary Fig. 14d) while the latter is the resonance of the core (Supplementary Fig. 14f). The peak positions are in agreement with the experimental peaks observed at 843 nm and 534.5 nm. The plasmon resonance wavelength is extremely sensitive to the precise dimension of gold nanostructure and a 1 nm change in the branch length, for example, can induce a peak shift exceeding 20 nm. Thus the slight discrepancy between the simulated and experimental peak wavelength is attributed to the small errors that may be present in estimating the precise dimensions of gold nanostructure.

AuNC-2 exhibits two resonances: one transverse resonance concentrated at the core and one longitudinal resonance with hot spots at the ends of the branches according to the

simulations (Supplementary Fig. 15). The transverse resonance is located at 520 nm and the longitudinal resonance at 810 nm. The simulation results faithfully reproduced the experimental spectra, which also showed two peaks (528.5 nm and 779.5 nm). The transverse resonance peak position agrees almost perfectly. The experimental longitudinal resonance is located at a shorter wavelength than the simulation. We attribute this difference to the slight overestimation of branch length in simulation. Reduction of the branch length by a few nm can make the peak position agree well.

AuNC-3 has less obvious symmetry and thus the simulations had to be done for a number of different orientations to identify all resonances supported by this structure. This modelling study revealed that AuNC-3 exhibits three resonances in all (Supplementary Fig. 16). One is the now familiar 520 nm resonance concentrated at the core (Supplementary Fig. 16c,d). The other two are resonances delocalised across the structure with hot spots at the tips of the three branches (Supplementary Fig. 16e). One of these two exhibits equal intensities at the tips of the three branches (Supplementary Fig. 16f) with a peak wavelength of 645 nm. The other has uneven distribution of electric field over the three branches (Supplementary Fig. 16g,h) and this resonance is located at 762 nm. The simulation results once again agree well with the experimental spectrum, which also show three peaks at 537 nm, 613.5 nm, and 749 nm.

For nanostars with four and five branches, their lack of symmetry requires a large number of simulations for multiple orientations. Since the simulations for the one-, two- and three-branched structures have already established the plasmon hybridizations between the core and the branches in agreement with experiments, we did not conduct further simulations for the four- and five-branched structures.

Supplementary References

1. Xia, Y., Xiong, Y., Lim, B. & Skrabalak, S. E. Shape-controlled synthesis of metal nanocrystals: simple chemistry meets complex physics? *Angew. Chem. Int. Ed.* **48,** 60-103 (2009).

2. Xiao, J. & Qi, L. Surfactant-assisted, shape-controlled synthesis of gold nanocrystals. *Nanoscale* **3,** 1383-1396 (2011).

3. Chiu, C. Y., Ruan, L. & Huang, Y. Biomolecular specificity controlled nanomaterial synthesis. *Chem. Soc. Rev.* **42,** 2512-2527 (2013).

4. Sun, W.*, et al.* Casting inorganic structures with DNA molds. *Science* **346,** 1258361 (2014).

5. Nehl, C. L., Liao, H. W. & Hafner, J. H. Optical properties of star-shaped gold nanoparticles. *Nano Lett.* **6,** 683-688 (2006).

6. Rodriguez-Lorenzo, L., Alvarez-Puebla, R. A., de Abajo, F. J. G. & Liz-Marzan, L. M. Surface enhanced raman scattering using star-shaped gold colloidal nanoparticles. *J. Phys. Chem. C* **114,** 7336-7340 (2010).

7. Yuan, H. K.*, et al.* Gold nanostars: surfactant-free synthesis, 3D modelling, and two-photon photoluminescence imaging. *Nanotechnology* **23,** 075102 (2012).

8. Wang, Z. D., Zhang, J. Q., Ekman, J. M., Kenis, P. J. A. & Lu, Y. DNA-mediated control of metal nanoparticle shape: one-pot synthesis and cellular uptake of highly stable and functional gold nanoflowers. *Nano Lett.* **10,** 1886-1891 (2010).

9. Nguyen, A. H., Ma, X. Y. & Sim, S. J. Gold nanostar based biosensor detects epigenetic alterations on promoter of real cells. *Biosens. Bioelectron.* **66,** 497-503 (2015).

10. Yuan, H. K.*, et al. In vivo* particle tracking and photothermal ablation using plasmon-resonant gold nanostars. *Nanomedicine.* **8,** 1355-1363 (2012).

11. Xie, J. P., Zhang, Q. B., Lee, J. Y. & Wang, D. I. C. The synthesis of SERS-active gold nanoflower tags for *in vivo* applications. *ACS Nano* **2,** 2473-2480 (2008).

12. Yuan, H., Fales, A. M. & Vo-Dinh, T. TAT peptide-functionalized gold nanostars: enhanced intracellular delivery and efficient NIR photothermal therapy using ultralow irradiance. *J. Am. Chem. Soc.* **134,** 11358-11361 (2012).

13. Mohanty, A., Garg, N. & Jin, R. C. A universal approach to the synthesis of noble metal nanodendrites and their catalytic properties. *Angew. Chem. Int. Ed.* **49,** 4962-4966 (2010).
